# Engineered Healing of Avascular Meniscus Tears by Stem Cell Recruitment

**DOI:** 10.1038/s41598-018-26545-8

**Published:** 2018-05-25

**Authors:** Solaiman Tarafder, Joseph Gulko, Kun Hee Sim, Jian Yang, James L. Cook, Chang H. Lee

**Affiliations:** 1 Regenerative Engineering Laboratory Columbia University Medical Center, 630W. 168 St. – VC12-230, New York, NY 10032 USA; 2 Department of Biomedical Engineering, The Pennsylvania State University, 205 Hallowell Building, University Park, Pennsylvania, PA 16802-4400 USA; 3 Thompson Laboratory for Regenerative Orthopaedics Missouri Orthopaedic institute, University of Missouri, 1100 Virginia Avenue, Columbia, Missouri, 65212 USA

## Abstract

Meniscus injuries are extremely common with approximately one million patients undergoing surgical treatment annually in the U.S. alone. Upon injury, the outer zone of the meniscus can be repaired and expected to functionally heal but tears in the inner avascular region are unlikely to heal. To date, no regenerative therapy has been proven successful for consistently promoting healing in inner-zone meniscus tears. Here, we show that controlled applications of connective tissue growth factor (CTGF) and transforming growth factor beta 3 (TGFβ3) can induce seamless healing of avascular meniscus tears by inducing recruitment and step-wise differentiation of synovial mesenchymal stem/progenitor cells (syMSCs). A short-term release of CTGF, a selected chemotactic and profibrogenic cue, successfully recruited syMSCs into the incision site and formed an integrated fibrous matrix. Sustain-released TGFβ3 then led to a remodeling of the intermediate fibrous matrix into fibrocartilaginous matrix, fully integrating incised meniscal tissues with improved functional properties. Our data may represent a novel clinically relevant strategy to improve healing of avascular meniscus tears by recruiting endogenous stem/progenitor cells.

## Introduction

Knee meniscus plays indispensable roles in joint congruence, shock absorption, and stress transmission^1^. Meniscus is characterized by its multiphase biochemical composition and complex structure^1^. The outer third region of meniscus is vascularized and constituted with dense fibrous matrix populated with fibroblast-like cells, whereas the inner third region is avascular cartilaginous tissue with chondrocyte-like cells. The middle region is fibrocartilaginous tissue with a mixed population of fibroblasts and chondrocytes^2^. Clinically, over one million patients undergo surgical repair or meniscectomy each year in the U.S.^3,4^. Tears in the vascularized outer third region of meniscus can typically be successfully surgically repaired by suturing. In contrast, tears in the inner avascular region, similar to articular cartilage, cannot be repaired due to its poor intrinsic healing capacity. As a result, these tears frequently progress to extend into the middle-third region, followed by meniscus deterioration^1,5,6^. Partial or total meniscectomy is often performed to alleviate symptoms caused by the irreparable meniscus injuries. However, meniscectomy significantly increases the incidence of osteoarthritis (OA) later in life by elevating joint contact stress. Approximately 50% of patients with meniscal injuries develop OA within 10 to 20 years of injury^1,5,6^. Allograft transplantation from cadavers may be considered after meniscectomy to prevent the increase in joint contact pressure, but is limited by donor shortage, sizing issues, subrejection immune responses, risk for extrusion, and potential for failure^1,5,6^. Despite the profound health care burden, no therapy exists to consistently induce healing of inner meniscus tears to date.

Various surgical and bioengineering approaches have been investigated to improve healing of avascular meniscus injuries. Fibrin clots and glues were applied to simply bond torn meniscus and have been reported to improve meniscus healing *in vitro* and *in vivo*^7,8^. Meniscal rasping, synovial flap implantation, and surgical induction of local blood supply have been applied to improve avascular meniscus healing^9^. Combination of dynamic loading and IL-1 enhanced integrative meniscal repair in an explant culture model^10^. Juvenile meniscus fragments implanted in avascular defects enhanced meniscus healing *in vitro*^11^. More recently, tissue engineering approaches have been applied for meniscus repair and healing^12^. Nanofiber-based scaffolds releasing collagenase were implemented to guide healing of avascular meniscus tears^12^. In another study, nanofiber-based scaffolds seeded with mesenchymal stem/progenitor cells (MSCs) enhanced meniscus healing *in vitro* and *in vivo*^13,14^. Three-armed and hyper-branched adhesive block copolymers were applied to improve healing of meniscus tears, which showed a modest enhancement in tissue healing and cell migration with support from TGFβ3 and collagenase treatment^15,16^. In other studies, injectable hydrogel derived from porcine meniscus extracellular matrix (ECM)^17^ and PLGA mesh scaffolds with pretreatment of platelet-rich plasma showed improved integration of meniscus explants^18^.

MSCs from bone marrow or synovium/synovial fluids, without a scaffold or tissue glue, also showed potential to improve healing of meniscus defects or tears^19–23^. Allogeneic synovial MSCs infused into 1.5-mm cylindrical meniscus defects in rabbits adhered to the injury site and apparently enhanced meniscal regeneration^20^. Similarly, infusion of autologous MSCs into sheep meniscal defects engrafted and improved healing^19^. Intra-articular injection of dual luciferase and LacZ tagged, synovial MSCs enhanced meniscus healing in the rat^21^. Recently, synovial MSCs delivery improved healing of longitudinal avascular tears in minipigs^22^. However, several experimental studies have shown an inconsistent outcome of meniscus healing by stem cell transplantation^23^. Previous studies have reported a limited recapitulation of biochemical composition and biomechanical properties, as well as incomplete integration of fibrocartilaginous matrix, which are critical goals towards functional success that have not been achieved to date.

**Figure 1:**
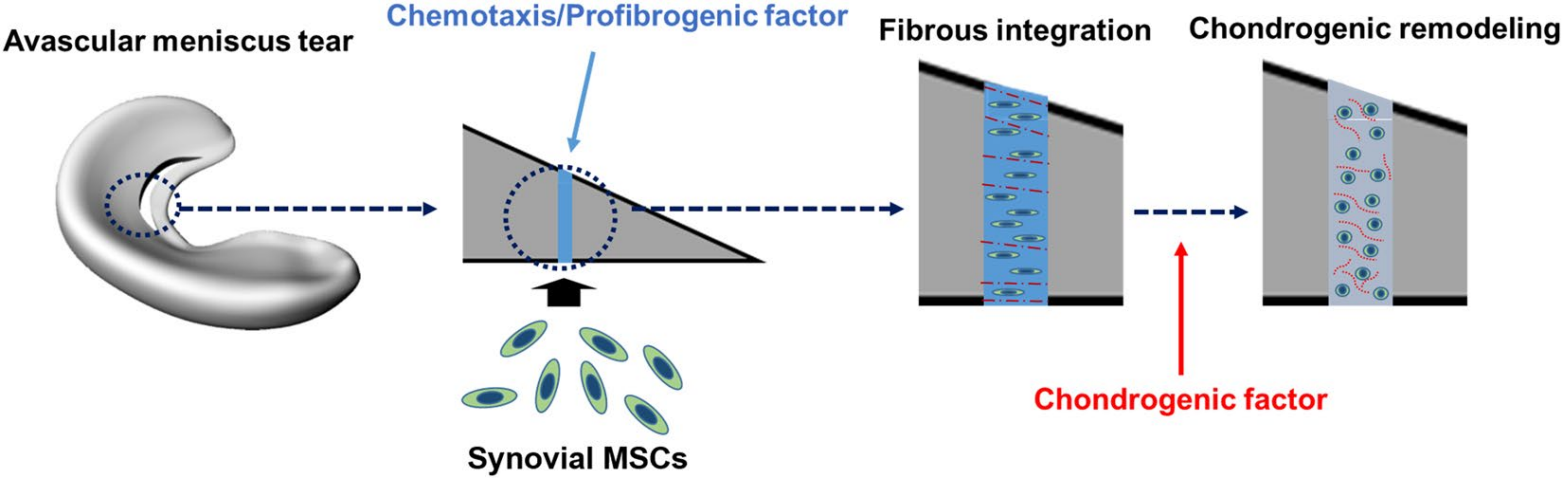
The novel strategy to induce seamless healing of inner meniscus tears by stem cell recruitment, followed by formation of intermediate fibrous integration and cartilaginous remodeling. Upon creation of a longitudinal tear in the avascular zone, chemotaxis/profibrogenic factor, CTGF, was applied to recruit syMSCs and to form fibrous integration. By applying chondrogenic factor, TGFβ3, the intermediate fibrous tissue is to be remodeled to region-specific fibrocartilaginous tissue.

Recently, we have demonstrated the step-wise differentiation of MSCs into fibrochondrocyte-like cells^24^, which is likely consistent with the phenotype transition observed in meniscal development^25^. At embryonic day 16 (E16), menisci are immature fibrous tissues predominantly expressing collagen type I that gradually transform into zone-specific fibrocartilage with increased expressions of collagen type II and aggrecan in the inner zone by 9 months of age^25^. Taking lessons from the meniscus development and our previous study, we here devised a novel approach to improve avascular meniscus healing by inducing recruitment and step-wise differentiation of synovial mesenchymal stem cells (syMSCs) (Fig. 1). Temporally controlled delivery of connective tissue growth factor (CTGF), a chemotactic/profibrogenic cue, and transforming growth factor beta 3 (TGFβ3), chondrogenic cue, successfully recruited syMSCs into the defect sites and formed integrated intermediate fibrous matrix, followed by fibrocartilaginous integration with functional restoration. Our data may represent a novel bioengineering approach to improve avascular meniscus healing with significant clinical impact.

## Results

### Sequential application of CTGF and TGFβ3 to guide meniscus healing by regulating recruitment of syMSCs and step-wise fibrocartilaginous differentiation

**Figure 2:**
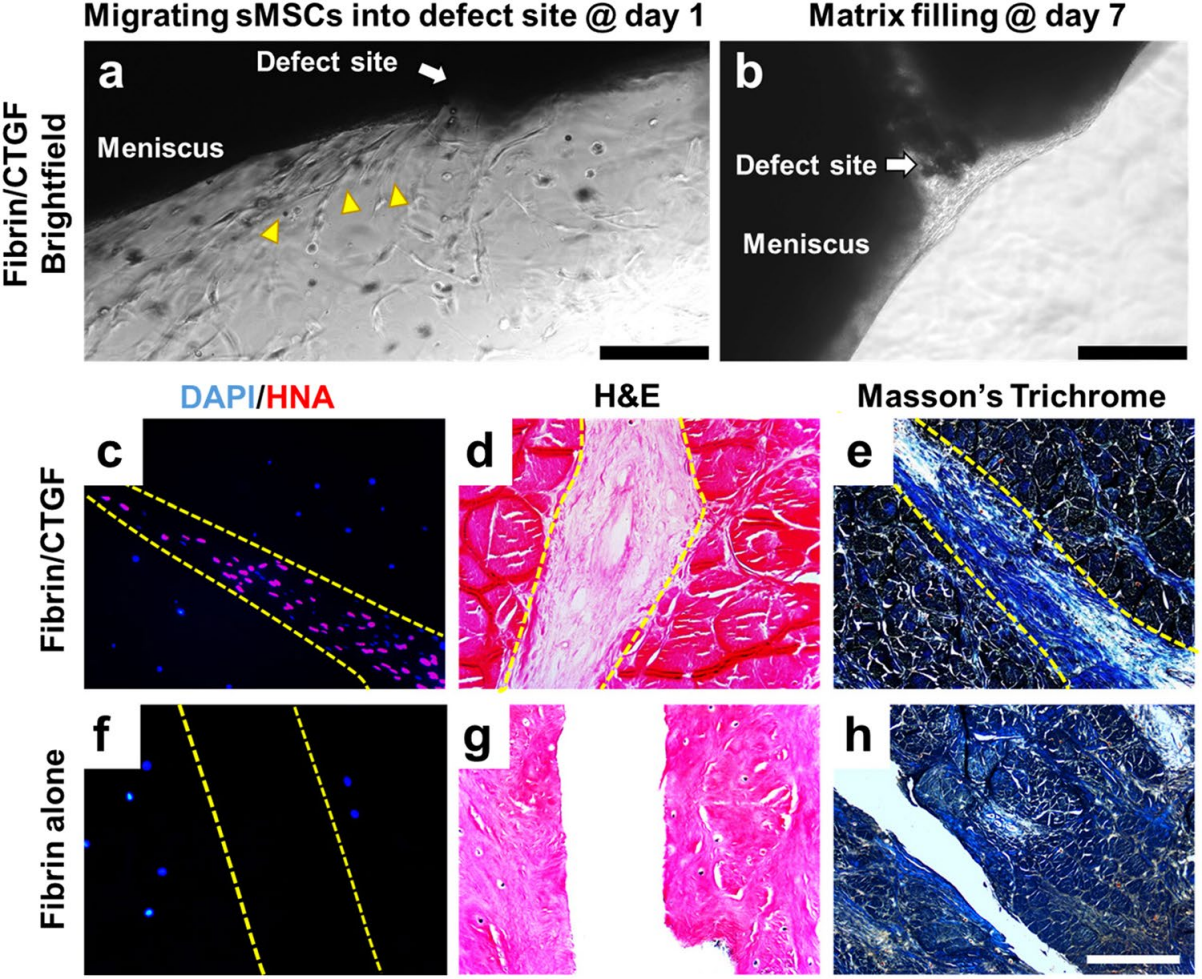
CTGF delivery via fibrin gel induced recruitment of syMSCs into meniscus defects at day 1 (**a**), followed by forming matrix by day 7 (**b**). HNA staining further showed the recruited syMSCs (**c**) in the intermediate fibrous tissues at 10 days, as confirmed by H&E and Masson’s Trichrome staining (**d**,**c**). With fibrin alone without CTGF, neither cell recruitment nor tissue integration was observed (**f**–**h**). Scale = 200 µm.

Explants from bovine menisci were used to study *in vitro* healing of avascular meniscus tears. Menisci were isolated from skeletally mature bovine knee joints provided by a local butcher shop. The inner 1/3 of total menisci were cut, and then 4–5 wedge-shaped explants were prepared from the inner 1/3 portion of meniscus by cutting in the radial direction. As shown in Fig. 1, a longitudinal incision was created using a surgical blade in the middle of the isolated inner third zone and then fibrin glue was applied to the incised site. Chemotactic and profibrogenic cue, CTGF (100 ng/mL), was delivered within the fibrin glue (50 mg/mL fibrinogen and 50 U/mL thrombin). Then, the meniscus explants were cultured on top of monolayer-cultured human synovial MSCs. The number of syMSCs significantly increases in synovial fluids after meniscus and cartilage injuries, thus being considered a clinically relevant endogenous cell source for meniscus regeneration *in vivo*^24,26,27^. After 10 days, 10 ng/mL of TGFβ3, a chondrogenic cue, was applied with chondrogenic supplements and cultured for 6 weeks. Starting at day 1, bright-field images revealed syMSCs migrating into the incision site where CTGF-loaded fibrin was delivered (Fig. 2a). The recruitment of syMSCs into incision sites with CTGF/fibrin was further confirmed by immunofluorescence with human nucleus antigen (HNA) at day 10 (Fig. 2c) in contrast to fibrin alone group, which showed no cell migration (Fig. 2f). By 10 days, tissue integration with fibrous matrix was established at the incision site with CTGF-loaded fibrin (Fig. 2b,d and e) in contrast to remaining gaps with fibrin alone (Fig. 2g,h), likely due to rapid degradation of fibrin.

**Figure 3:**
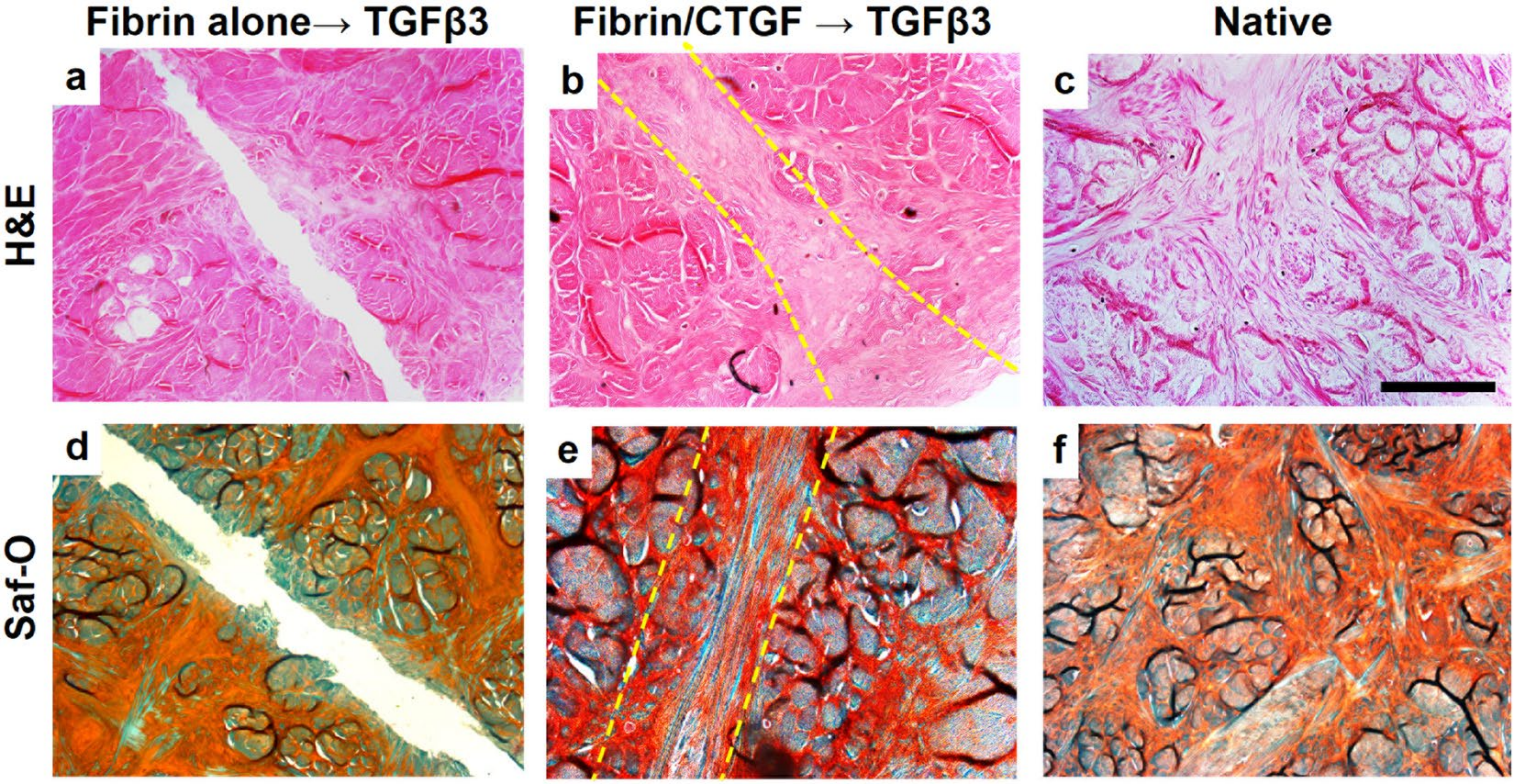
Fibrocartilaginous healing of inner meniscus by sequential application of CTGF and TGFβ3 by syMSC recruitment after 6 wks. H&E (**a**–**c**) and Saf-O (**d**–**f)** staining showed integrated fibrocartilaginous healing of avascular meniscus tears by fibrin/CTGF followed by TGFβ3 (**b**,**e**) as compared to fibrin alone, followed by TGFβ3 (**a**,**d**). Dotted line indicates the healing zone. Native (**c**,**f**) indicates intact meniscal tissues at avascular zone. Scale = 200 µm.

Upon TGFβ3 treatment, the intermediate fibrous matrix appeared to be remodeled into fibrocartilaginous tissue, fully integrating incised meniscal tissues (Fig. 3b,e) similar to native (Fig. 3f)^28,29^. The healed meniscal tissues showed native-like fibrocartilaginous phenotype with rounded chondrocyte-like cells (Fig. 3b,c,e and f). However, fibrin glue alone, followed by TGFβ3 treatment failed to undergo tissue integration or remodeling (Fig. 3a,d).

### Avascular meniscus healing by a single application of CTGF-loaded fibrin glue mixed with TGFβ3-encapsulated PLGA µS

**Figure 4:**
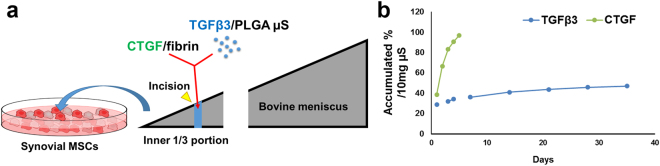
Application of CTGF (100 ng/mL)-loaded fibrin glue mixed with PLGA µS-encapsulating TGFβ3 (10 mg/1 mL) to the avascular meniscus healing model (**a**). *In vitro* release profile (**b**) shows the fast release of CTGF within 5 days and sustained release of TGFβ3 from PLGA µS.

**Figure 5:**
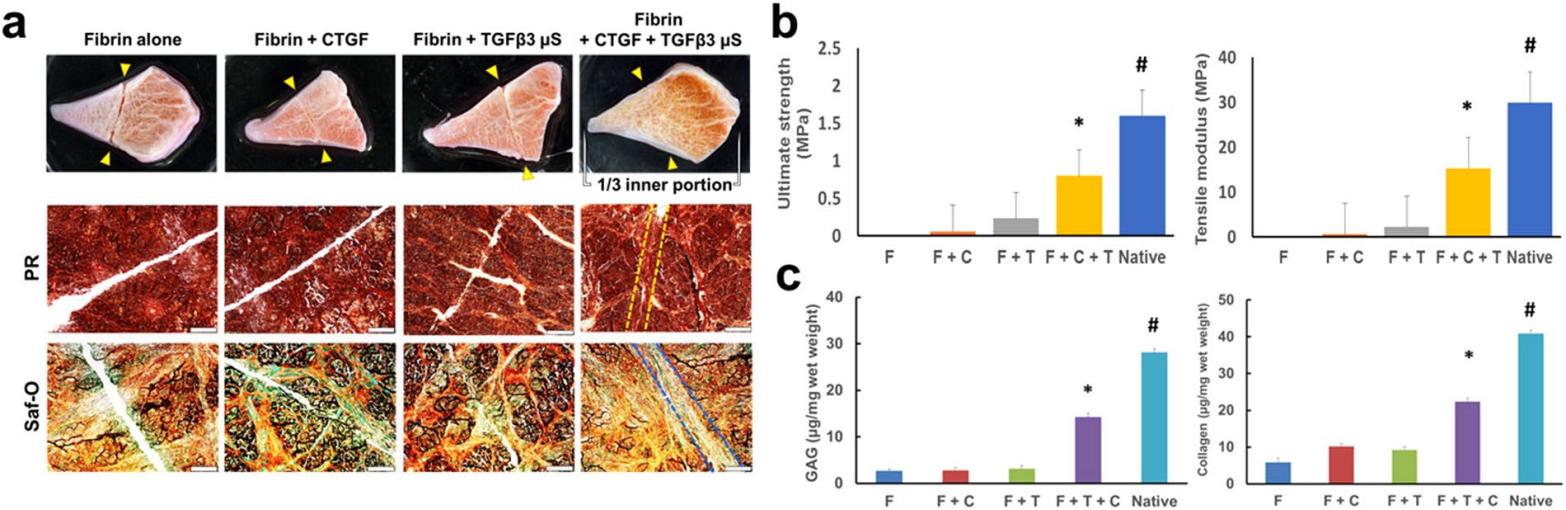
Avascular meniscus healing improved by CTGF-loaded fibrin glue mixed with PLGA µS-encapsulating TGFβ3, while fibrin alone, fibrin with CTGF and fibrin with TGFβ3-µS failed to lead to integration of the torn meniscus tissues (**a**). Pull-out strengths and tensile moduli were significantly higher in the meniscus healed by CTGF-fibrin glue with TGFβ3-µS (**b**). GAG and total collagen contents from 500 µm-width strip samples including the defect site were significantly higher with CTGF-fibrin glue with TGFβ3-µS (PR: Picrosirius Red, Saf-O: Safranin O/Fast Green, F: fibrin alone, F + C: fibrin + CTGF, F + T: fibrin + TGFβ3 µS, F + C + T: fibrin + CTGF + TGFβ3 µS). n = 6 per group, *p < 0.05 compared to all other groups; ^#^p < 0.05 compared to F + C + T. Scale = 200 µm.

**Figure 6:**
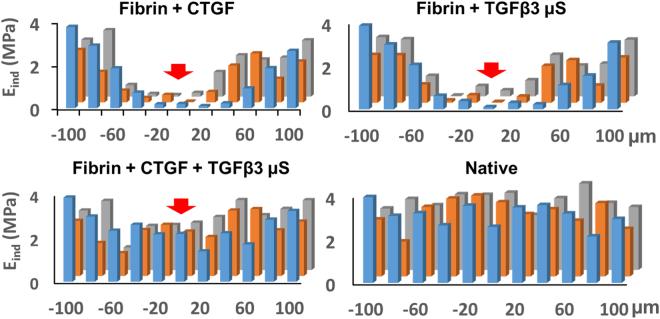
Indentation moduli (E_in_) measured at every 20 µm distance from the defect site of meniscus explants (−100 to 100 µm in x-axis; −20 to +20 µm in y-axis). After 6 wks, Fibrin + CTGF and Fibrin + TGFβ3 show poor E_in_ in the healing regions. Fibrin + CTGF + TGFβ3 enhanced the continuity of E_in_, corresponding to that of native menisci. Red arrow indicates the tear site. The different color bars show E_in_ at different positions on the distal-proximal axis (in parallel to direction incision) (Blue: 0 µm, Orange: 20 µm, and Gray: 40 µm).

In consideration of preclinical translational modeling and eventual clinical application of the timely controlled treatment of CTGF and TGFβ3 for avascular meniscus healing, we applied CTGF-loaded fibrin glue mixed with poly(lactic-co-glycolic acids) (PLGA) microspheres (µS) (50 ± 13 µm of dia.)-encapsulated with TGFβ3 in our meniscus explant model. TGFβ3 encapsulated in PLGA µS were prepared as per our established methods^24,30^. This approach enabled a single injection into meniscus injuries that provides short-term release of CTGF, followed by sustained release of TGFβ3 (Fig. 4). PLGA µS-encapsulating TGFβ3 (2.5 µg per 250 mg PLGA) were prepared by the double-emulsion technique as per our prior work^24,31^. A total of 100 ng/mL CTGF was loaded in 50 µL of thrombin (50 U/mL) mixed with 10 mg of PLGA µS-encapsulating TGFβ3. Then, CTGF-loaded thrombin with TGFβ3-µS was co-injected with 50 mg/mL fibrinogen into incised meniscal tissues using FibriJet^®^ dual-injector with a blending applicator. The explants were then cultured in media with 1:1 mixed fibrogenic and chondrogenic supplements following our existing protocol^24^. *In vitro* release study by ELISA showed that CTGF was completely released within 5 days and TGFβ3 was released over 36 days (Fig. 4b). The meniscus explants harvested at 6 wks showed a fully integrated fibrocartilaginous healing when fibrin loaded with CTGF and TGFβ3-µS was applied (Fig. 5a,b). However, fibrin alone, fibrin with CTGF, and fibrin with TGFβ3 resulted in a poor integration or a remaining gap in the incised meniscus tissues (Fig. 5a,b). The ultimate pull-out strength and tensile modulus of the healed menisci by CTGF-loaded fibrin with TGFβ3-µS were significantly higher than fibrin alone, fibrin with CTGF, and fibrin with TGFβ3-µS groups (Fig. 5b). Similarly, glycosaminoglycans (GAG) and collagen contents of the healed meniscus by CTGF-loaded fibrin with TGFβ3-µS were significantly higher than fibrin alone, fibrin with CTGF, and fibrin with TGFβ3-µS groups (Fig. 5c). Modulus mapping with nanoindentation was performed to examine a spatial variance in the mechanical properties of healed meniscus (PIUMA™ nano-indenter; Optics11, Amsterdam, The Netherlands). Fibrin loaded with CTGF and TGFβ3-µS enhanced the continuity of indentation modulus (E_in_), corresponding to that of native menisci (Fig. 6). These findings indicate that profibrogenic cue or chondrogenic cue alone is not sufficient to induce integrated healing of avascular meniscus tears, and the sequentially controlled formation of intermediate fibrous integration followed by cartilaginous matrix remodeling is pivotal to guide integrative fibrocartilaginous meniscus healing.

### *In vivo* healing of avascular meniscus defects by endogenous syMSCs

**Figure 7:**
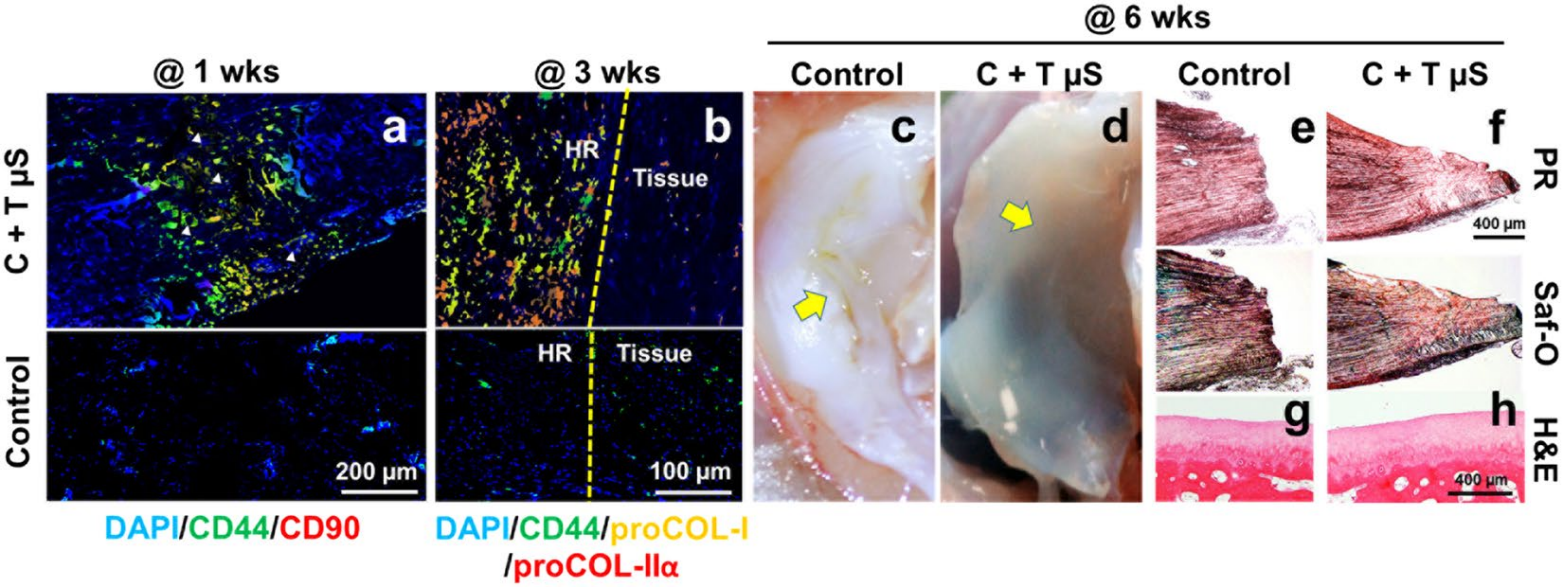
Rabbit meniscus healing *in vivo* by application of fibrin-loaded with CTGF and TGFβ3 µS. By 1 wk post-op, the recruitment of CD44^+^/CD90^+^ cells into the surgically created meniscus tears was observed with delivery of CTGF and TGFβ3 µS in contrast to control (**a**) (arrow heads indicate remaining PLGA µS). The recruited CD44^+^/CD90^+^ cells undergo differentiation into proCOL-I^+^/proCOL-Iiα^+^ fibrochondrocyte-like cells by 3 wks (**b**) (HR: healing region). CTGF-loaded fibrin with TGFβ3 µS (C + T µS) successfully enhanced avascular meniscus healing (**c**,**d**) (Yellow arrows indicate the tear sites), supported by PR (**e**) and Saf-O staining (**f**). There were no noticeable damage on the articular surfaces both with the control (**g**) and C + T µS groups (**h**).

Upon confirming *in vitro* efficacy, we applied the CTGF-loaded fibrin with TGFβ3-µS for *in vivo* meniscus healing. Critical sized longitudinal tears (5 mm) were surgically created in the inner 1/3 zone of skeletally mature NZW rabbits (n = 10 total) and then CTGF-loaded fibrin with TGFβ3-µS was applied. After 1 week, recruitment of CD44^+^/CD90^+^ syMSC-like cells^27^ into the meniscus tears was confirmed by immunofluorescence (Fig. 7a). The recruited CD44^+^/CD90^+^ syMSC-like cells underwent differentiation into proCOL-I^+^/proCOL-IIα^+^ fibrochondrocyte-like cells by 3 weeks (Fig. 7b). At 6 weeks post-op, delivery of CTGF-loaded fibrin with TGFβ3-µS improved meniscus healing (Fig. 7d) in contrast to remaining gaps in control groups with fibrin alone (Fig. 7c). Control group tears treated with fibrin alone failed to integrate meniscal tissues (Fig. 7e). In contrast, the CTGF-loaded fibrin with TGFβ3-µS resulted in integrated healing of meniscus with picrosirius red (PR)^+^ and safranin O (Saf-O)^+^ staining fibrocartilaginous tissue (Fig. 7f).

## Discussion

Our findings suggest a novel and efficient strategy to induce healing of avascular meniscus tears by recruitment and step-wise differentiation of endogenous stem/progenitor cells. Short-term (<5 days) delivery of CTGF in fibrin glue successfully induced recruitment of syMSCs into surgically created defects and then formed intermediate fibrous matrix integrating the incised meniscal tissues. Then, sustain-released TGFβ3 from PLGA µS over 6 weeks led to fibrocartilaginous healing of avascular meniscus tears with significantly improvements in biochemical and functional properties. Our second-generation approach using CTGF-loaded fibrin glue mixed with TGFβ3-µS enables controlled delivery of CTGF and TGFβ3 by a single injection, which may represent a minimally invasive approach to induce meniscus healing in patients. In addition, the present study demonstrated the feasibility of an *in situ* tissue engineering approach for meniscus healing by endogenous stem/progenitor cells instead of transplantation of *ex vivo* cultured cells. Despite being a valid approach, cell transplantation has encountered crucial barriers in therapeutic translation, including immune rejection, pathogen transmission, potential tumorigenesis, issues associated with packaging, storage, and shipping, and difficulties in clinical adoption and regulatory approval^26,32^. Thus, our *in situ* tissue engineering approach by endogenous stem/progenitor cells may hold realistic translational potential for treatment of avascular meniscus injuries by overcoming the limitations related to cell transplantation.

Similar to articular cartilage, the inner zone of meniscus will rarely heal, primarily due to lack of blood supply, immobility of resident cells, and limited matrix turnover^33^. Although various tissue engineering approaches using stem cells, growth factors, and/or physical stimulation showed potential to engineer tissue grafts to replace the injured/damaged meniscus, the functional integration between the engineered grafts and host tissues remains as one the most challenging goals^5,13,24,34–36^. Consistently, our data showed that TGFβ3 alone is not sufficient enough to induce an integrated healing of meniscus tears despite its eminent function in chondrogenic/fibrochondrogenic differentiation of MSCs^24,26,37^. Thus, it is essential to incorporate a process to guide the tissue integration with tissue engineering of fibrocartilaginous tissues for functional meniscus healing. Previous studies applied collagenase or matrix metalloproteinase (MMP) combined with chondrogenic cues (e.g. TGFβ3) to improve integration of avascular meniscal tissues with engineered fibrocartilaginous tissues^12,37,38^. In this study, we achieved the integrative healing of avascular meniscus tears by applying a novel bioengineering approach based on meniscus development. Inspired by the notable phenotype transition of meniscus from a fibrous tissue to a regionally distributed fibrocartilaginous tissue in the process of development and maturation, we incorporated a treatment with a potent profibrogenic cue, CTGF, to engineer intermediate fibrous matrix prior to prolonged treatment with TGFβ3. In contrast to cartilaginous matrix, fibrous matrix is a default filler for tissue defects, providing a suitable biochemical and physical environment for cell migration/infiltration and tissue integration and remodeling^39^. Consistently, our data demonstrated induction of intermediate fibrous integration by CTGF in avascular meniscus healing, likely representing an efficient strategy to improve integration of avascular tissues such as meniscus, articular cartilage, and temporomandibular disc joint.

Despite promising *in vitro* and *in vivo* advances for avascular meniscus healing, our study has several limitations. First, only longitudinal meniscus tears were tested in our explant and *in vivo* models. The longitudinal tear is the most prevalent (~46%) among all the tear patterns in human patients (radial: ~7%, Flap: ~4.5%, complex: ~13%)^6,40^, and a longitudinal tear in human menisci over 10 mm in length is considered unstable and in need of repair to prevent progression to a degenerative defect^6^. Thus, our 5-mm longitudinal tears in rabbit meniscus are considered a critical sized defect with clinical relevance. However, the longitudinal tear is mechanically more stable than the other types of tears. Thus, follow-up studies will investigate the efficacy of our *in situ* tissue engineering approaches for healing more challenging meniscus defects such as radial, flap, and complex tears^6,40^. Second, rabbits, despite being a valid animal model for meniscus healing, have limitations with respect to small joint size, use of clinical surgical techniques, and capabilities for functional outcome measurements^41^. Given the small tissue size and narrow healing zone in rabbit meniscus, the multi-scale mechanical tests performed in bovine meniscus explant were not applicable to rabbit tissues. Accordingly, a well-established large animal model (e.g. canine) will be adopted for follow-up studies to test the long-term *in vivo* efficacy of our *in situ* tissue engineering approach for avascular meniscus healing. Structural properties and functional restoration of meniscus associated with the load-bearing and joint function will be comprehensively investigated in the follow-up large animal studies. Third, our study lacked understanding of *in vivo* release kinetics of CTGF and TGFβ3 which is likely different from *in vitro*. Recent technical emergences have potential to label various materials with fluorescence dye or particles through various approaches including but not limited to chemical modification and physical encapsulation^42,43^. Our follow-up study will consider such labeling technique to perform imaging-based tracking of *in vivo* degradation and associated release of growth factors. Follow-up study will also address the potential participation of endogenous cell sources besides synovial cells. Lastly, fibrin gel, despite being an efficient experimental tool as a growth factor carrier, has poor mechanical stability, weak wet adhesion strength, and rapid degradation rate that may not be suitable for long-term *in vivo* application. Although fibrin gel was sufficient as a carrier for CTGF and TGFβ3-µS for meniscus healing in bovine explants and the rabbit model, advanced bio-adhesives^44,45^ with improved mechanical stability, adhesion strength, and injectability will need to be considered for follow-up large animal studies.

In conclusion, our findings demonstrated the integrated fibrocartilaginous healing of avascular meniscus tears by a temporal regulation of stem cell recruitment, fibrogenic integration, and fibrocartilaginous matrix remodeling. These data serve as an important foundation for developing a regenerative therapy for avascular meniscus injuries. Delivering a consistent method for healing of avascular meniscus tears would improve outcomes and reduce the incidence and severity of degenerative osteoarthritis in patients. Accordingly, our *in situ* tissue engineering approach using CTGF-loaded hydrogel with TGFβ3-µS holds significant promise for advancing healthcare for this large and growing problem.

## Methods

### Meniscus explant for avascular healing by syMSCs recruitment

A meniscus explant model was used to study *in vitro* healing of avascular meniscus tears. Menisci were isolated from skeletally mature (18–24 months old) bovine knee joints from a local butcher shop. The isolated menisci were rinsed with 10X antibiotics (5 mins; 2 times), 1X antibiotics (5 mins; 2 times), and then washed in PBS. The inner third zone of menisci were cut and prepared as wedge-shaped tissue explants in a thickness of 2–3 mm. Then full-thickness longitudinal incisions was made in the middle of the inner third zone, and fibrin glue loaded with or without 100 ng/ml CTGF (BioVendor, Asheville, NC) was applied to glue the incised tissues. Briefly, 50 mg/mL fibrinogen (Sigma-Aldrich, St. Louis, MO) and 50 U/mL thrombin (Sigma-Aldrich, St. Louis, MO) with or without CTGF were co-injected at a total volume of 50 µl in between the incised tissue surfaces using FibriJet^®^ dual-injector with a blending applicator (Nordson Micromedics, Westlake, OH). Then the meniscus explants were placed on the monolayer cultured P2 - P3 human syMSCs, established from our previous work^24^. A mixture of 1:1 fibrogenic induction supplement (50 μg/mL ascorbic acids) and chondrogenic induction supplements (1% 1 × ITS + 1 solution, 100 μg/ml sodium pyruvate, 50 μg/ml L-ascorbic Acid 2-phosphate, 40 μg/ml L-proline, and 0.1 μM dexamethasone) were applied during the explant culture. By 10 days, recruitment of syMSCs and formation of fibrous matrix were analyzed using bright-field microscopy, immunofluorescence for human nucleus antigen (HNA), H&E and Masson’s Trichrome staining, as per our prior methods^24,26,31,46^. Then chondrogenic cue, TGFβ3 (R&D Systems, Minneapolis, MN**)**, was applied at a concentration of 10 ng/mL for 6 wks. Fibrocartilaginous tissue integration was evaluated by H&E and Saf-O/Fast Green staining. GAG and collagen assays were performed with 500 µm-thick tissue samples containing the healed region, as per our prior methods^26,31,46^.

### CTGF-loaded fibrin gel with TGFβ3 encapsulated in PLGA microspheres

In order to apply a sequential release of CTGF and TGFβ3 via a single injection for *in vivo* application, we prepared fibrin gel loaded with CTGF and PLGA microspheres (µS)-encapsulating TGFβ3 (2.5 µg per 250 mg PLGA). PLGA (66,000-107,000 Mw) with a PLA/PGA ratio of 75:25 was purchased from Sigma (St. Louis, MO). PLGA µS encapsulating recombinant human TGFβ3 were prepared by a modified double-emulsion technique^24,47^, a well-established control-delivery vehicle demonstrating preserved bioactivity of growth factors. Briefly, 500 mg PLGA was dissolved into 5 mL chloroform followed by adding 250 µL of diluted TGFβ3. This solution was then emulsified (primary emulsion) by ultrasonicating for 5 minutes to reduce the size of µS^30^. The primary emulsion (w/o) was then added to 10 mL 4% (w/v) PVA (poly vinyl alcohol) solution to form the second emulsion (w/o/w) by 2 minutes ultrasonication followed by 1 minute vortexing. This double emulsion solution was then added to 250 mL of 0.3% PVA solution followed by continuous stirring for 2 hours to evaporate the solvent. Finally, the microspheres (µS) were filtered, washed with DI water, resuspended in DI water and then lyophilized. For a measurement of release rates, CTGF-loaded fibrin gel with PLGA µS-encapsulating TGFβ3 were incubated at 37 °C with gentle agitation for 5 wks in PBS and 1% BSA for CTGF and TGFβ3, respectively. At selected time points, incubation media was collected and concentration of CTGF and TGFβ3 were measured using ELISA as per our previous works^24,31^. For application in explant meniscus healing model and *in vivo* meniscus tears, fibrinogen was co-injected with thrombin containing CTGF (100 ng/ml; final concentration) and PLGA µS encapsulating TGFβ3 (10 mg µS/ml; final dose) into the incision sites at a total volume of 50 µl and then allowed for gelation for 1–2 mins. The doses of CTGF and TGFβ3 were adopted from our pilot study (data not shown) and previous works^24^.

### Tensile tests

Following a well-established testing protocol for meniscus explants^19^, samples for the pull-out tests were prepared using a cryotome as 500~600 µm in thickness and a width of 1 mm as per previous works^48^. Upon mounting with tensile jigs in an isotonic saline bath at RT, a 0.02-N tare load was applied to the samples and then the samples will be elongated at 10%/min until failure. From the force vs. elongation curve, the ultimate strength and tensile modulus were obtained. Briefly, the tensile modulus was calculated as the slope of stress (force/cross-sectional area) vs. strain (displacement/initial length), and the ultimate strength represent maximum load divided by cross-sectional area. All pull-out tests werer performed using Electroforce^®^ BioDynamics^®^ system (Bose Corp., Eden Prairie, MN).

### Modulus and friction mapping and surface congruency

Nanoindentation experiments was conducted using a PIUMA™ nano-indenter (Optics11, Amsterdam, The Netherlands) with a probe of 1-μm with the sample loaded to a maximum force of 10 mN. All nanoindentation tests were carried out on unfixed and unstained tissue sections^49^. A series of indentations were performed to determine the indentation modulus (E_in_) across a healed region at every 20 μm distance from the original defect site, using the embedded high-precision mobile X-Y stage.

### *In vivo* model for avascular meniscus healing

All the animal procedures for inner meniscus healing followed a protocol approved by the Institutional Animal Care and Use Committee at Columbia University, carried out in accordance with relevant guidelines and regulations. Skeletally mature NZW rabbits were used for the *in vivo* study (2.5–3.0 kg; n = 10; fibrin alone and fibrin/CTGF/TGFβ3-µS groups; two harvested at 1wk; two harvested at 3 wks; six animals harvested at 6 wks). Under general anesthesia, the bilateral knee joints were incised through the medial parapatellar approach. A vertical incision (about 5 mm long) along the longitudinal axis of the medial meniscus was made with a No. 12 knife in the avascular area of the anterior half of the meniscus. Then fibrin gel (20 µl) supplemented with 100 ng/ml CTGF and 10 mg/mL TGFβ3-µS was applied to the incised area. At 1, 3, and 6 wks post-op, the animals were euthanized and meniscus tissues were harvested for analyses.

### Immunofluorescence

Following our prior methods^31,50^, immunofluorescence was performed to image tissue sections using monoclonal antibodies and isotype-matched Alexa Fluor^®^ secondary antibodies, with nucleus labeling with DAPI. All the tissue sections were made in 5-µm thickness and the antigen retrieval procedures were performed following the manufacturer’s protocols. Human nuclear antigen (HNA) (ab191181, Abcam, Cambridge, MA), CD44 (MA4400, Life Technologies, Grand Island, NY), CD90 (ab226, Abcam, Cambridge, MA), proCOL-I (C7510-11F, United States Biological, Salem, MA), and/or proCOL-IIα (ABIN1385152, Antibodies-Online) were co-labeled with multiple fluorescent secondary antibodies to track recruitment and differentiation of endogenous syMSCs. All images were acquired using an inverted fluorescence microscope (Olympus IX73, Waltham, MA).

### Statistical analysis

For all the quantitative data, following confirmation of normal data distribution, one-way analysis of variance (ANOVA) with post-hoc Tukey HSD tests were used with p value of 0.05. Sample sizes for all quantitative data were determined by power analysis with one-way ANOVA using an a level of 0.05, power of 0.8, and effect size of 1.50 chosen to assess matrix synthesis, gene expressions, and mechanical properties in the regenerated meniscus tissues and controls upon verification of normal data distribution.
